# Self-Assembly in Biosilicification and Biotemplated Silica Materials

**DOI:** 10.3390/nano4030792

**Published:** 2014-09-04

**Authors:** Francisco M. Fernandes, Thibaud Coradin, Carole Aimé

**Affiliations:** Sorbonne Universités, UPMC Univ Paris 06, CNRS, Collège de France, UMR 7574, Chimie de la Matière Condensée de Paris, F-75005 Paris, France; E-Mails: francisco.fernandes@upmc.fr (F.M.F.); thibaud.coradin@upmc.fr (T.C.)

**Keywords:** self-assembly, silica, biomineralization, biomimetism

## Abstract

During evolution, living organisms have learned to design biomolecules exhibiting self-assembly properties to build-up materials with complex organizations. This is particularly evidenced by the delicate siliceous structures of diatoms and sponges. These structures have been considered as inspiration sources for the preparation of nanoscale and nanostructured silica-based materials templated by the self-assembled natural or biomimetic molecules. These templates range from short peptides to large viruses, leading to biohybrid objects with a wide variety of dimensions, shapes and organization. A more recent strategy based on the integration of biological self-assembly as the driving force of silica nanoparticles organization offers new perspectives to elaborate highly-tunable, biofunctional nanocomposites.

## 1. Introduction

Biomineralization encompasses all biological pathways leading to the formation of a condensed inorganic phase [1]. As such, it includes both biologically-induced and biologically-controlled precipitation. In the first situation, the formation of the inorganic solid is adventitious, arising from non-specific interactions between mineral sources and components or metabolic products of living organisms. In the second case, mineralization occurs through a complex cellular pathway by the production of specific molecules that control the composition, shape, organization and, ultimately, the properties and functions of the inorganic phase. Because such a control implies a strong intimacy between the inorganic system and the biomolecules involved in mineralization control, resulting biominerals are in fact composite materials, where the organic fraction can range from less than 1 wt% (urchin spikes) to 50 wt% (crab shells) [2]. These molecules have three main functions: confinement (to control mineral particle size), activation (to control mineral sources concentrations) and templating (to control particle morphology) [3]. Ultimately, these functions also contribute to the spatial organization of the particles within the composite structure. Thus, one of the key properties of biomineralizing molecules is their self-assembly ability, either as single components or cooperatively with others.

These self-assembly processes have largely contributed to the merging of experts in material science, earth science, biology and medicine in order to achieve a better understanding of biomineralization pathways. In chemistry, one of the main motivations was related to the possibility to mimic or get inspired by biological routes for the design of novel synthetic strategies and/or new objects [4]. In this context, the possibility to control the formation and organization of inorganic particles from the nano- to the macroscale using soft matter principles raised great hopes for the development of chemically-diverse, structurally-complex and bio-responsive devices [5,6,7].

Silica-based materials occupy a specific position in this field. Silicon is the most abundant element in soils, a fraction of which originates from the sedimentation and diagenesis of biogenic silica. This is related to the fact that biosilicification occurs in many living organisms, being terrestrial (higher plants) or aquatic (diatoms, radiolarians, sponges) [8]. Biogenic silica materials often exhibit astonishing morphologies that have no synthetic equivalent. This reflects the malleability of silica in a hydrated amorphous form, as obtained by condensation of silicic acid in ambient conditions [9]. Such a malleability is in fact one of the major factors explaining the wide use of silica as the main component of many synthetic materials. Therefore it does not come as a surprise that biomimetic or bio-inspired routes to silica-based materials have been widely developed.

Several reviews covering the principles and applications of these approaches are available [10,11,12]. In this paper, we choose to focus on one specific aspect of biosilicification processes and biomimetic/bioinspired silica synthesis that is the role and use of biomolecular self-assembly for the controlled formation of nanoscale and nanostructured materials. After a brief presentation of the current knowledge of self-organization pathways in diatoms and sponges, we describe how natural or nature-like molecules, from peptides to virus, can be used as templates for silica formation. As a perspective, we introduce an alternative strategy to combine biological molecules and silica via a bottom-up approach that opens a wide range of possibilities for the design of functional bionanocomposites.

## 2. Natural Self-Assembled Silicified Structures 

### 2.1. Diatoms

**Figure 1:**
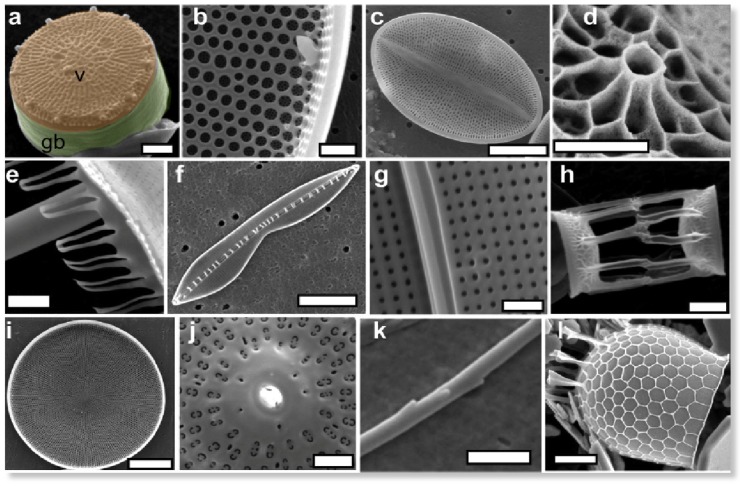
Morphological diversity in diatoms frustule. Colored areas in (**a**) show valve (v) and girdle band (gb). Scale bar (**a**,**k**) 1 µm; (**b**) 5 μm; (**c**,**f**,**l**) 10 μm; (**d**) 500 nm; (**e**,**g**,**h**,**j**) 2 μm; (i) 50 μm. Copyright 2008 American Chemical Society. Reprinted with permission from reference [13].

The biosilicification processes occurring in diatoms can be followed during cell division [13]. The mother cell is surrounded by a frustule consisting two lids (or valves) of slightly different diameters that are fitted together via a ring structure (the girdle band) (Figure 1). Cell division occurs inside this box, resulting in two daughters’ cells, each associated with one of the lids. At this stage, a specific vesicle, called the silica deposition vesicle (SDV), is formed. Silicic acid is up taken by the cells, transported into the SDV and is condensed into a new valve whose dimensions fit those of the mother cell’s lid. At this stage, the young valve is released by exocytosis and a new girdle band is formed, allowing for complete separation of the two daughter cells.

The composition of the frustule has been widely investigated over the last 30 years. Carbohydrates, proteins, lipids and other biomolecules have been identified with various degrees of intimacy with the silica network [14]. Such diversity can be put in parallel with the structural complexity of the frustules. For instance, in centric diatoms, the valves are formed of densely-packed silica nanoparticles with size varying from 50 to 150 nm that constitute the walls of an array of pores with an hexagonal organization and a lattice constant in the μm scale [15]. In contrast, the silica structure of the girdle band consists of a square array of pores with a periodicity in submicronic range [16]. Over the whole frustule volume, the pore size can vary from the μm range down to a few tens of nm. Noticeably, the pore size gradient from the external to the internal side of the frustule can be from the largest to smallest pores or reverse depending on the considered species [17].

Facing the chemical diversity and structural complexity of biosilica as formed by diatoms, several groups have attempted to identify some common components or mechanisms that could explain, at least partially, how frustule is elaborated [11]. At this time, it is possible to consider three different phenomena: (i) the induction of silica condensation; (ii) the control of silica particle size and packing; (iii) the formation of the frustule patterns (*i.e*., porous structure). As shown afterwards, these processes can occur simultaneously and involve several molecules acting cooperatively.

Two main classes of biomolecules involved in diatom biosilicification have been described: phosphorylated proteins and long-chain polyamines. The first type of proteins, termed silaffins, are polycationic polypeptides, rich in lysine amino acids exhibiting post-translational modifications consisting of 6 to 11 repeats of a *N*-methyl-propylamine unit [18]. In addition, they contain eight phosphate groups resulting from the post-translational phosphorylation of serine amino acids. The modified lysines introduce a high number of positive charges and hydrophobic groups while phosphorylation introduces negative charges. Altogether, these modifications confer a zwitterionic character to the proteins [19]. This is especially the case of the native silaffin-1A (natSil-1A) that has been shown to activate the condensation of pre-hydrolyzed silicon alkoxides *in vitro* in mild conditions (neutral pH and ambient temperature and pressure) [20]. Such an activation is related to the ability of natSil-1 to self-organize into an assembly of *ca*. 700 molecules interacting together via intermolecular electrostatic interactions. Noticeably, dephosphorylation of natSil-1A inhibits its self-assembly ability and therefore its influence on silica formation. However, further addition of phosphate anions restores its silica precipitation activity. In contrast, native silaffin-2 (natSil-2) and silaffin-3 exhibit long polypeptide moieties and post-translational modifications that make them strongly negative [21]. Therefore, they have no effect on silica condensation. However, when mixed with natSil-1A, they exert some regulatory effect on silica morphogenesis. Depending on the natSil-1A/natSil-2 ratio, silica nanospheres, nanoporous silica blocks and interconnected particles could be obtained *in vitro*. Here again, electrostatic interactions together with the hydrophobic character of the proteins play a major role on proteins self-assembly and activation of silica formation, ultimately influencing silica morphology at the microscale. The same principles apply when considering the reactivity of long-chain polyamines (LCPA). These molecules consist of linear oligopropyleneimine chains connected with propylenediamine, putrescine or spermidine [22]. It was found that, in the presence of anions such as phosphates or sulfates, they undergo a microscopic phase separation phenomenon. These microdroplets could act as templates for the formation of silica spheres, whose size was strictly dictated by the phosphate concentration. Alternatively, it was possible to generate different silica structures by mixing silaffins and polyamines [23]. However, it is important to question the relevance of these *in vitro* models involving isolated biomolecules and synthetic sources of silica for the elucidation of *in vivo* mechanisms. Indeed these studies converge in identifying the importance of the presence of positively-charged group and of the self-assembly properties of the biomolecules on their biosilicifying ability. However, it is important to point out that these experiments are performed using supersaturated silicic acid solutions near neutral pH that spontaneously lead to silica precipitation. This contrasts with silicification conditions in diatoms, *i.e*., a yet unknown organically-stabilized silicon forms in slightly acidic conditions [13]. Therefore, if these model studies have been particularly useful to provide guidelines for the design of biomimetic and bio-inspired systems, as described in the following section, they still leave many unanswered questions about the cellular mechanisms of silicification.

In particular, the question of the overall morphological control of the frustule at the macroscale remains open. The key role of the diatom cytoskeleton on silica organization has been recently evidenced [24]. This work demonstrates that the frustule morphogenesis process involves a top-down mechanism where the actin network is responsible for the three-dimensional positioning of the SDVs, ultimately controlling silica deposition patterns. Two other important studies have suggested that additional molecules can be involved in silica structuration. It has been shown that fibrous networks of chitin are present in the valves, whose organization is very close to the organization of silica spheres [25]. In the girdle bands, a new family of proteins, termed cingulins, has been identified that self-assemble to form nanopatterned microrings and could act as template for silica formation [16]. Interestingly, in both cases, these templates do not favor silica condensation. However, addition of LCPA allows the precipitation of silica on the cingulin-based macroscale templates. This enlightens again the need for a combination of molecules to achieve a hierarchical organization of silica.

### 2.2. Sponges

Silicified spicules are needle-like extracellular secretions mainly found in two classes of sponges: Hexactinellid and Demosponges [26,27]. In Hexactinellid, so-called “giant” spicules are found up to 3 m in length and 8.5 mm in diameter. Smaller spicules, 450 μm in length and 5 μm in diameter, were identified in Demosponges. The hybrid architecture of these spicules consists of an axial filament surrounded by an alternation of organic layers with lamellae of silica nanoparticles. A gradient in silicification extent is observed from the core to the surface of the spicule [28]. The silica structure close to the filament is dense and the bioorganic component is hardly visualized whereas the alternation of mineral-organic layers is more evident for more external lamellae. Such a layered structure is indeed indicative of the sequential course of the biosilicification process.

**Figure 2:**
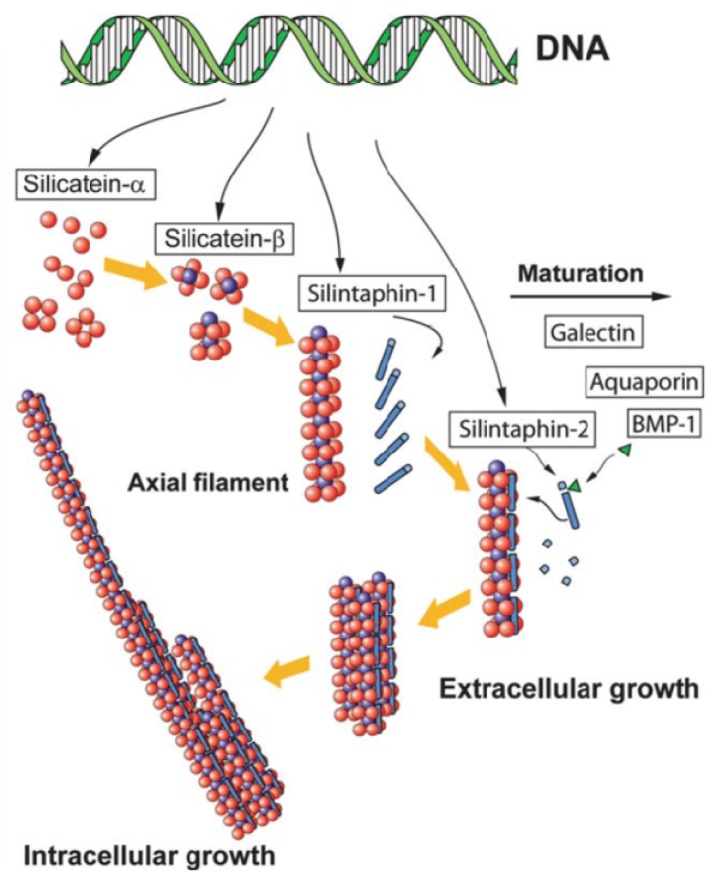
The self-assembly process of the organic layers in siliceous spicules. Copyright 2012 The Royal Society of Chemistry. Reprinted with permission from reference [29].

Based on the recent works by Müller and co-workers, a precise mechanism of spicule formation is available (Figure 2) [29]. Specific cells, termed sclerocytes, synthesize a protein, silicatein, present in three isoforms α, β, and γ that self-assemble intracellularly. The α form is predominant and exhibits a catalytic activity towards silicon alkoxide hydrolysis. Silicatein α has a high peptide sequence homology to the cathepsin protease [30]. Both enzymes have six cysteine residues forming intramolecular disulfides so that the tertiary structures of the two proteins are expected to be very similar. However, when considering the active site, the histidine and asparagine amino acids are present in the two proteins whereas the cysteine residue of cathepsin is replaced by a serine amino acid in silicatein-α. Therefore the active site of silicatein α is similar to that of serine proteases (trypsin, chymotrypsin) where a hydrogen bond exists between the imidazole nitrogen of histidine and the OH group of serine. This enhances the nucleophilicity of the hydroxyl oxygen, favoring its nucleophilic attack on the silicon atom, whereas the proton interacting with nitrogen can be transferred to the silicon substituent, the two processes catalyzing the hydrolysis of silicon alkoxide. However neither silicatein α nor the association of the three silicatein isoforms exert any specific control on silica morphology because other constituents are necessary to achieve a suitable self-organization.

These constituents include galectin as well as two proteins termed silintaphins [31]. These proteins are present both during the intracellular synthesis of the axial filament and during its extracellular extension and in the organic layers located between the silica lamellae. Their interplay is rather complex and may involve Ca^2+^ binding events. As a matter of fact, compared to protein self-assembly in diatoms, much less is known about the molecular mechanisms of interactions between the different constituents of the spicule filaments. Nevertheless, *in vitro* studies using polyethyleneglycol addition to the axial filament showed that it leads to the enhancement of its mechanical properties, suggesting the key role of hydrogen bond in its organization.

In terms of silica structuration, fiber diffraction studies showed that the proteins adopt a mesoporous organization within the axial filament that was replicated by mineral network [32]. Hence, as for diatoms, a combination of template self-assembly and silica formation activation should be involved in spicule organization. Another interesting aspect of silica morphological control is related to the observation of the evolution of mineral density through a decrease in particle size and in porosity upon spicule ageing [33]. This was associated with the presence of aquaporins in the membrane of cells that can be involved in water expulsion during syneresis.

## 3. Silicification of Self-Assembled Biomolecules

### 3.1. Self-Assembled Peptides

The R5 peptide is a member of a family of short amino acid sequences obtained after maturation of a protein Sil-1p, a precursor of Sil-1 [18]. Site-directed mutagenesis studies of this short 19-amino-acid peptide has evidenced the presence of a critical *C*-terminus motif that directs the self-assembly of the peptide. This results in a high local concentration of amines providing an active site for silica precipitation. Hence, as for silaffins and polyamines, a combination of electrostatic interactions and hydrogen bonds leads to R5 supramolecular structures that act both as catalysts and templates for silica formation *in vitro* at neutral pH [34]. This reactivity was used for the formation of carbon nanotube/silica using R5-containing multifunctional peptides identified from a combinatorial phage display library.

Synthetic peptides that share similarities to the silica-nucleating proteins can also be used to design peptide-based scaffolds capable of inducing and controlling silica formation. A nice example of this approach is the use of the lanreotide octapeptide incorporating two primary amine moieties together with tyrosine (for hydrogen bonding) and cysteine (for intramolecular disulfide bond formation) [35]. When put in water, lanreotide forms supramolecular gels consisting of peptide nanotubes with diameter *ca*. 25 nm and wall thickness *ca*. 2 nm. Upon addition of silicon alkoxide on its surface, the gel progressively dissociates while hybrid silica/lanreotide tubes *ca*. 30 nm in diameter are formed. Interestingly, these tubes have a double wall structure, consisting of silica-peptide-silica layers. Overall it can be suggested that the gel acts as a reservoir for peptides that are released as individual molecules into the alkoxide solution. A co-assembly of lanreotide and silica occurs leading to the depletion of the supernatant solution in free peptide that drives further the gel dissolution.

**Figure 3:**
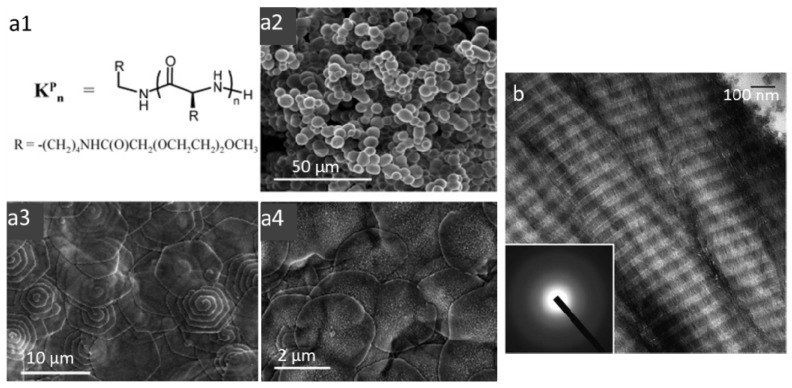
(**a1**) Chemical structure of PLL (K^p^_n_) and Scanning Electron Microscopy (SEM) images of (**a2**) K^p^_56_ spheres, (**a3**,**a4**) K^p^_264_ and K^p^_400_ hexagonal plates. Copyright 2006 American Chemical Society. Reprinted with permission from reference [38]. (**b**) Unstained Scanning Electron Microscopy (TEM) images of silicified collagen with the cross-banding architecture of fibrillar collagen. Inset: selected area electron diffraction reveals the amorphous nature of the infiltrated minerals. Copyright 2011 Wiley. Reprinted with permission from reference [41].

Homo and block copolypeptides bearing basic amino acids, lysine, histidine and arginine should in principle constitute efficient catalysts for silica precipitation. As described extensively for poly-l-lysine homopolymers (PLL) [36], the high density of positively-charged groups is able to concentrate negatively-charged silicic acids and promote silica condensation [37]. However, the ability of these homopolymers to self-assemble in a significant manner is quite limited. The most successful approach along this line is the use of long-chain PLLs that can adopt an α-helical conformation, that is able to template the formation of silica platelets (Figure 3a) [38]. In contrast, short PLLs could favor silica condensation but led to featureless silica nanospheres because of the absence of self-organization. Based on the silaffin structure, lysine-containing block co-polypeptides (Lys-b-AA) have been designed and evaluated as silica templates [39,40]. A wide variety of silica morphologies, including platelets, rods, columns and hollow spheres, could be achieved by varying the nature of the hydrophobic block (AA = Cysteine, Leucine, Glycine, Alanine). This morphological diversity results from the balance between the lysine-silica interactions and the polypeptide self-assembly properties deriving from their hydrophobic character. Introduction of an aromatic amino acid, such as phenylalanine, allows the induction of π-stacking interactions, leading to vesicles or micelles that can be used to obtain silica particles of tunable size.

The case of ultrashort [Lysine-(Isoleucine)_3_] peptides is worth being discussed here because their self-assembly should ultimately result in supramolecular structures similar to the (Lys-b-AA) copolypeptide systems [42]. These peptides have the ability to form long nanotubes that were sufficiently stable for direct templating of silica from tetraethyl orthosilicate (TEOS) near neutral pH. Further control of silica morphology could be achieved by using a mixture of silicon alkoxides. Thus, the co-condensation of 3 aminopropyltriethoxysilane (APTES) and TEOS at different ratios allowed the generation of silica fibers and string-of-beads [43]. Such morphological variations can be attributed to the presence of additional amine groups that can influence the peptide self-assembly. 

### 3.2. Proteins

Based on the above-discussed principles of biosilicification, any protein that is positively-charged and exhibit some self-assembly properties can constitute a template for silica formation. A typical example is Lysozyme that exhibits an isolelectric point (pI) of 10.5 and a high content of hydroxyl- and imidazole-containing amino acid. This protein was shown to produce composite nanospheres with varying size and interconnectivity as a function of pH, precursor, and lysozyme concentration [44]. From these data, it was suggested that silicon sources influence lysozyme aggregation that in turn templates silica formation.

These results enlighten the possibility for silica or its condensation reaction to influence the self-assembly process of the biological template, as also illustrated with collagen-silica biomaterials [45]. Type I collagen, the main protein of mammalian tissues, is synthesized as a triple helix, *ca.* 1.5 nm in diameter and 300 nm in length. It has a pI of *ca.* 7 and can therefore be stabilized as a sol in acidic media. In these conditions, highly concentrated collagen solutions (>60 mg·mL^−1^) can exhibit a liquid crystal phase behavior [46]. At lower concentration, an increase in pH leads to the self-assembly of triple helices into fibrils themselves aggregating into fibers ultimately forming hydrogels. Earlier studies have shown that it is possible to silicify pre-formed collagen liquid crystalline phases and fibrils *via* vapor phase or solution approach, respectively [47,48]. A more complex approach was recently described based on the infiltration of a poly-aspartic acid (PAA)-enriched collagen sponges with choline-stabilized silicic acid [41] (Figure 3b). The choline derivative allows reducing the reactivity of the silicon source, allowing for slow diffusion and mineralization of the collagen network. Attempts were also made to perform collagen self-assembly and silica condensation simultaneously by neutralization of an acidic mixture of triple helices and silicates. It was found that a low amount of silicic acid favors the fibrillogenesis process but that increasing amount of the silica source could perturb and ultimately hinder collagen self-assembly [49]. This effect was attributed to electrostatic interactions between silicic acid and collagen. SEM observations indicated that at intermediate silica content, collagen fibers were organized in rope-like structures that could originate from the electrostatic repulsion between silica-coated fibrils that prevent their alignment. Interestingly, such structures showed better mechanical properties than pure hydrogels due to the presence of the silica network but lower compatibility for skin cells as they could not access the collagen surface required for their optimal adhesion.

Further insights on the underlying mechanisms were gained during the preparation of gelatin-based hybrid materials. Gelatin is obtained by denaturation of collagen, in particular by an acidic treatment leading to type A gelatin with a pI of *ca.* 8. This protein is well-known to form thermo-induced gels below 37 °C due to partial reformation of the initial collagen triple helices. When silicate species were added to a gelatin sol at pH 7, a hybrid precipitate was formed with an ill-defined morphology [50]. In contrast, when performed at pH 5, fibers, platelets and particles of various sizes were obtained depending on gelatin and silicate concentration. Later on, the precipitation of silica on gelatin films was also studied, demonstrating that the strength of the gelatin network, an indication of its chemical stability that is related to the extent of collagen denaturation, also influenced the silica particle size [51]. These data strengthen the idea that the silica templating process depends on the balance between gelatin-gelatin, silicate-silicate and gelatin-silicate interactions, all of which acting simultaneously and cooperatively in optimal conditions.

### 3.3. Polysaccharides

Similarly to proteins, polysaccharides can, in principle, be used as template for silica formation. Cellulose is particularly attractive for that purpose due to its ability to form chiral liquid crystal phase and was used as a template to design mesoporous materials [52]. However, very few polysaccharides are polycations so that biomimetic silicification approaches were mainly described using chitosan. Chitosan is obtained by deacetylation of chitin, a polymer of *N*-acetylglucosamine that is widely distributed in fungi, insects and marine animals. Interestingly, despite its overall positive charge, chitosan does not activate silica formation, which can be related to the large amine-amine interdistance along the polymer backbone together with the absence of self-assembly. Therefore most of the literature describes the unspecific silicification of pre-formed chitosan hydrogels or microspheres [53]. Nevertheless, it was found that soluble chitosan has an effect on the aggregation of silica particles formed at pH 5.6, suggesting that they can act as bridging molecules [54].

As seen in the case of diatoms, a combination of a precipitating molecule with a structuring agent may allow achieving a control of silica formation kinetics and of its morphology. For instance, the combination of alginate, a negatively-charged polysaccharide, with gelatin, allows to fine tune the precipitation of the silica [55]. This idea of a dual templating approach was prolonged in a mixture of acacia gum and gelatin [56]. These two natural macromolecules self-assemble into coacervates that were used as coating for oil droplets. Addition of TEOS leads to silica deposition on the oil surface, leading to core-shell particles.

### 3.4. Lipids

Amphiphile molecules possess both a polar hydrophilic group and a non-polar hydrophobic moiety, providing the key for self-organization. Many biomolecules exhibit amphiphilic behavior, including peptides and proteins. With respect to the other sections of this review, we will restrict ourselves on rather conventional amphiphiles that bear a charged or uncharged polar head connected to a hydrophobic tail, typically a hydrocarbon chain. Beside micelles and vesicles, amphiphilic molecules can self-assemble into a rich diversity of nanostructures such as fibers, ribbons, helices, and tubes. This has attracted a strong interest of materials chemists for the soft templating of nanostructured sol-gel materials with various morphologies and in mild experimental conditions [57]. Two different approaches can be distinguished. The first one drives the synthesis of mesoporous materials. This commonly implies amphiphile micelles (or alternatively more concentrated liquid crystalline phases) and silica precursors, usually TMOS (tetramethylorthosilicate) or TEOS, interacting cooperatively in a non-covalent way. The silica precursors adsorb on the micelles, where the condensation of the silicates takes place. Afterwards, the amphiphile is removed by calcination or solvent extraction, leading to a mesoporous material with a templated pore structure. One key point of this strategy is the possibility to tune the symmetry, pore size and wall thickness playing with the starting amphiphiles to adapt the materials, notably for catalysis, drug delivery, adsorption and separation processes. The synthesis of mesoporous materials has been thoroughly and recently reviewed by many authors [57,58,59], and we will focus here on the second approach that uses amphiphiles to direct the synthesis of individualized single objects with well-defined sizes and shapes.

**Figure 4:**
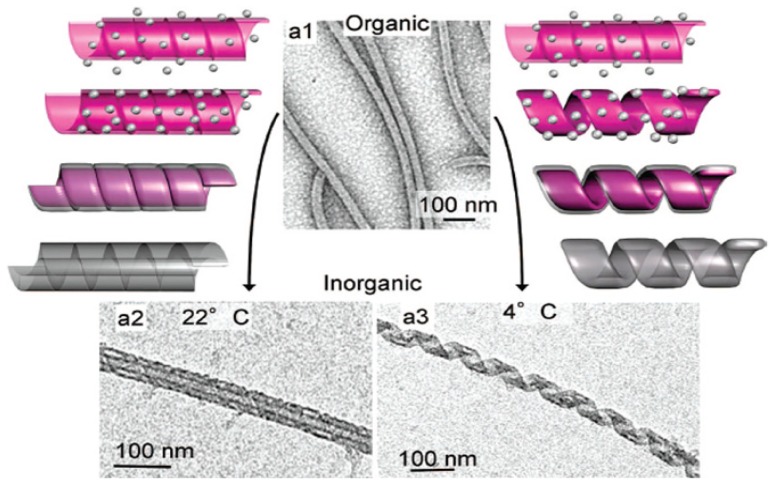
TEM photos showing the effects of the transcription temperature on (**a1**) gemini surfactants organogels having tubular morphology; (**a2**) silica nanotubes at 22 °C; and (**a3**) silica nanohelices at 4 °C. Copyright 2008 American Chemical Society. Reprinted with permission from reference [66].

Besides micelles and vesicles that have been used to template hollow spheres and vesicles of silica [60], more sophisticated nanostructures have been produced from amphiphiles assemblies [59]. As such, ribbons, helices, and tubes have been used to template silica through the sol-gel process, towards applications in nanotechnology, asymmetric catalysts, and optical devices. In 1998, Shinkai’s group [61] reported the first successful utilization of organogels as templates for the preparation of hollow silica fibers. A couple of years later, they show how chirality of the inorganic material could be controlled playing with the organic template. They reported on the sol-gel transcription of helically-structured silica in chiral diaminocyclohexane organogels based on electrostatic interactions. They found that right- and left-handed helical silica structures could be created from TEOS polymerization depending on the pure enantiomer used [62]. Silica nanotubes have also been transcribed from amphiphiles assemblies [63], for example by using the sol-gel reaction with TEOS and an organic template based on laurylamine hydrochloride (C_12_H_25_NH_2_.HCl) [64]. Interestingly, this surfactant-assisted templating mechanism allowed tuning the nanotube length and diameter. Finally, Oda and co-workers reported on a single amphiphilic system that gives rise to a wide diversity of morphologies. They used a cationic bis-quaternary ammonium gemini surfactants C_2_H_4_-1,2-((CH_3_)_2_N^+^C_16_H_33_)_2_ with tartrate as a counter-ion providing multiple ways to finely tune organic nanometric chiral assemblies (ribbons, helices and tubules) [65]. Those nanometric ribbons and tubules could be transcribed to inorganic nanostructures using sol-gel process with TEOS in absence of catalyst or cosolvent (Figure 4) [66]. By controlling parameters such as reagent concentration and kinetics of both organic assembly and inorganic polycondensation (ageing time, temperature) the selective formation of individualized twisted, helical or tubular silica was achieved. More interestingly, competition between silica precursors and tartrate counter-anions for condensation onto cationic membranes of gemini surfactants influence the morphology of the silica chiral ribbons. For example, this allowed the formation of silica twisted ribbons that were not accessible with the organic templates at room temperature. This shows that the dynamic and versatile nature of the organic template considerably enhances the tunability of inorganic materials with rich polymorphisms.

### 3.5. Virus

Viruses are mesmerizing molecular superstructures composed of clearly distinctive structural parts. The viral particles, called virions, can be described, from their interior to their outer surface, as a succession of genetic material, protein-based capsid and, in some cases, an outer lipid-based envelope. Hence, the interactions associated with the silicification of virus may have been described by the affinity of silica precursors towards the virion outer components, proteins or lipids. Such interactions have been discussed in the preceding sections. However, the significance of associating viruses and silica can hardly be described on the sole basis of these interactions. Rather, the outstanding aspects of silica-virus hybrids spring from the viral capsids’ form as well as the virus functional potential. In summary, virus form and function!

**Figure 5:**
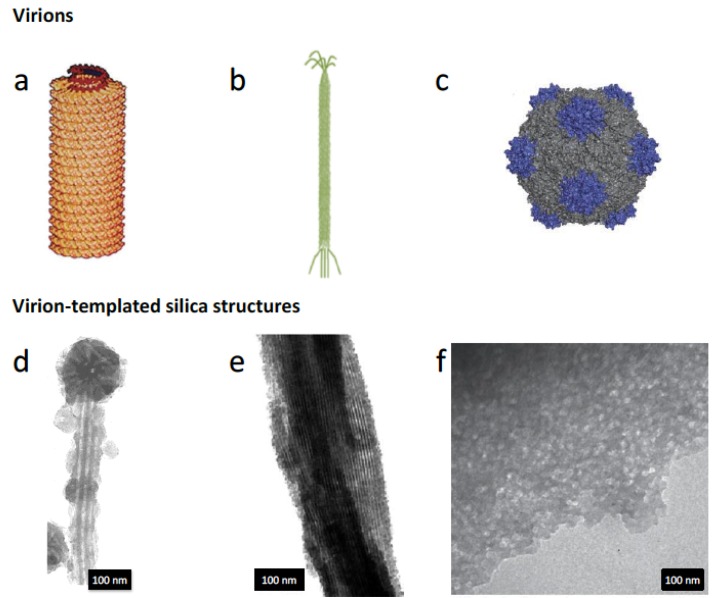
Macromolecular structures of different virus used for silica templating: (**a**) Tobacco mosaic virus; (**b**) T4 bacteriophage; and (**c**) Cowpea mosaic virus. TEM photos shown in (**d**–**f**) display the silica morphology obtained after calcination of TMV-, T4 bacteriophage- and CPMV-silica hybrids, respectively. (**a**,**c**,**f**) is reproduced with permission of Springer from reference [73]; (**b**) is reproduced from reference [74] with permission of The Royal Society of Chemistry; (**d**) and (**e**) are reproduced from references [71] and [72], respectively, with permission from Wiley.

From the helical 300 nm long rod-shape Tobacco Mosaic Virus (TMV) [67] (Figure 5a) to the 28 nm icosahedral Cowpea Mosaic Virus (CPMV) [68] (Figure 5c), viral capsids present a wide variety of regular geometric forms that place them as ideal candidates as building blocks in materials chemistry. One of the first examples of such approach concerns the application of Cowpea Chlorotic Mottle Virus (CCMV) capsid as a template for the production of metal oxide nanoparticles [69]. The empty virion capsid was used as a reactor to mineralize single crystalline domains of paratungstate and decavanadate compounds grown inside the virion cage, and thus corresponding to the capsid internal morphology. Unsurprisingly, the interactions between the clearly-defined geometrical cages of virions have also been explored as a template for silicification. Mann and co-workers have addressed the question in two successive reports [70,71]. In the first work, the authors have evaluated several mineral precursors and mineralization conditions in presence of TMV and observed the resulting morphologies [70], evidencing that several reactions could take place at the surface of TMV virions. When TEOS was hydrolyzed and later condensed in presence of a low concentration of TMV particles suspended in an acidic aqueous solution, the virion capsids were covered by a thin amorphous silica deposit. Moreover, the silicified virion particles aligned in an end-to-end arrangement, yielding very high aspect ratio silicified particles. Since the silicification seemed to form a very thin coating of the capsids, the authors further explored the liquid-crystal properties of TMV particles along with silica precursors to form mesostructured silica-virus materials [71] (Figure 5d). Other high aspect-ratio virions such as M13 bacteriophage (Figure 5b) have been used as templates for the development of silica structures. Mao and co-workers [72] have prepared silica fibers presenting hexagonally-packed aligned pores (Figure 5e) by the co-condensation of TEOS and APTES in presence of suspended wild-type M13 bacteriophage. Icosahedral virions such as CPMV have also been used to tailor the porosity of silica structures. Niu and co-workers [73] have followed the same approach used with TMV and M13 bacteriophages to prepare porous CPMV-silica composite materials presenting, after calcination, N_2_-BET surfaces up to 220 m^2^·g^−1^ (Figure 5f). These examples reflect the potential of virus particles to act as extremely well-defined templates for the shaping of the internal morphology of porous silica materials. However, this approach considers the virions as mere sacrificial templates to be eliminated upon calcination.

The association of virus and silica has also been explored in more functional approaches. One of the first works on silica-virus constructs that actually considered the virus functionality has been reported by Chen and co-workers [75]. The authors focused on the ability of amino-functionalized silica to capture and release two types of bacteriophages, MS-2 and PRD-1. To achieve maximal capture efficiency, the authors have tested different amine group-bearing silanes, from APTES to lupamin, a linear polyvinylamine silane. According to the authors the charged ammonium groups were responsible for the high capture efficiency. The capture efficiency observed for PRD-1 bacteriophage ranged from 9% up to 99% using bare silica and lupamin-derived silica, respectively. The ability of the amino-derived silica to release the viruses was assessed in the presence of different eluents achieving a maximum of 69.5% for PRD-1. Despite the lack of mechanistic information, the association between amino-functionalized silica and bacteriophages is, in all likelihood, dependent on the surface charge of both entities and thus a non-specific interaction of electrostatic nature. Another example where the association of silica and viruses seems promising relates to controlled delivery applications. Oncolytic viruses such as adenovirus are expected to bring dramatic improvement in cancer treatment. However, effective delivery of the virions to the targeted area is an important bottleneck. Kangasniemi and co-workers [76] have tackled this problem by encapsulating replication-competent adenovirus in silica materials to establish the effect of the association between silica and the virion on the delivery. Although the morphological characterization of the virus-silica construct is uncertain, the virus embedded in the silica shell had prominent antitumor activity in a pancreatic cancer model. Moreover, the silica shell seemed to trigger a less significant immune response than that observed for the virus particles alone.

Besides the biomedical applications of virus-silica constructs, other aspects have been addressed. For instance, Laidler* et al*. [77,78] probed the protective influence of silica coating on the thermostability of T4 bacteriophages. The formation of an outer silica shell had decreased the virus infectivity. However, upon desilicification, the viral activity could be recovered up to 10% of the initial titer. The consequence of the reversible deactivation of the virus particles upon silica shell formation is strongly related to the increased thermal and dessication tolerance of silicified viruses. The formation of an inorganic outer shell around the virion could explain the dispersion of effective virus particles by stratospheric winds or their existence in hot springs. In fact, the evidence for silicified virus-like particles has been found in extreme thermal environments such as hot springs [79].

In summary, although sparingly explored, the association of virions with silica precursors presents various benefits, from the formation of ordered mesoporous materials to the development of functional adenovirus delivery systems.

## 4. Biological Self-Assembly of Silica Nanostructures: A Change of Perspective

This review has so far discussed silica formation on pre-formed biological (or bio-inspired) templates, or cooperatively with them. Another way of designing silica-biomolecules composites consists of driving the organization of pre-formed silica structures based on the dynamic self-assembly of biomolecules, without the need of a template. Silica particles are widely used in various fields because of their ease of preparation, the possibility to control their size, their tunable and rich surface chemistry and because of the biocompatibility of silica. In the last section of this review, we aim at opening the discussion towards the exploration of biological self-assemblies as a tool to direct the organization of silica particles, an approach that we believe will give rise to a new family of silica-based biocomposites with unique properties.

Organizing silica particles *via* the self-assembly of biomolecules requires the careful functionalization of the particles with the biomolecule of interest, prior to self-assembly. In this respect, the interfaces between bio-organic elements and inorganic nanoscale materials have gathered a lot of interest, especially concerning *peptides and proteins* interacting with silica particles [80,81]. However, the outcomes of those works are often directed towards the elaboration of drug release systems [82]. Another approach of interest was reported by Lisdat and co-workers describing the immobilization of enzymes by layer-by-layer deposition [83]. In this work, they demonstrate the formation of silica particles/cytochrome c multilayer assemblies on electrodes using carboxy-modified silica nanoparticles as an artificial matrix. Alternatively, Gilchrist and co-workers reported on the use of streptavidin-functionalized silica particles as platforms for the self-assembly of supported lipid bilayers [84]. These two works provide good examples of the assembly of biomolecules onto silica particles surfaces. Very interestingly, they focus on the influence of the size and surface chemistry of silica nanoparticles on the construction of artificial systems based on natural proteins or lipids assemblies. However, they do not attempt to further direct silica particles organization.

**Figure 6:**
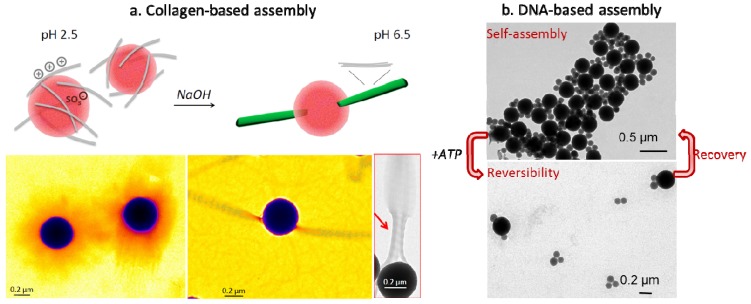
(**a**) Collagen adsorption and fibrillogenesis from sulfonated-modified silica nano-surfaces, as shown by TEM. Copyright 2014 The Royal Society of Chemistry. Reprinted with permission from reference [86]. (**b**) TEM photos of the reversible assembly of DNA-based silica particles networks. Copyright 20143 The Royal Society of Chemistry. Adapted with permission from reference [87].

One approach based on the bottom-up construction of silica networks from protein self-assembly can be found by the association of silica particles with collagen. Our group has first engineered a hybrid building block consisting of silica particles coated with collagen that possess intrinsic self-assembling properties (Figure 6). Collagen could be electrostatically bound as a triple helix to silica prior triggering of its self-assembly into large, well-organized fibrils [85]. A surface-mediated growth of the collagen fibrils could be evidenced that was attributed to the fact that although the protein was initially confined on the particle, it preserves some mobility allowing for its subsequent self-assembly [86].

Beside proteins, nucleic acids have become one of the most promising tools in biotechnologies. Indeed, self-assembled structures can be engineered by programming DNA sequences. As a result, DNA has been widely used to direct colloid assembly based on molecular recognition. However, those works mainly involve gold particles because their assembly-disassembly are correlated with modification in their optical properties, providing basis for the design of biosensors [88,89]. The immobilization of oligonucleotides on silica has been reviewed more than a decade ago but these studies focused on the design of single objects for the detection of DNA through hybridization [90]. More recently, DNA-based assembly of silica particles was reported [87,91]. In this work, Wu and co-workers used two populations of silica nanoparticles grafted with complementary DNA sequences, one of which bearing a central sequence, called aptamer, that is sensitive to adenosine triphosphate (ATP). The resulting bionanocomposite network could be disassembled upon ATP addition, converting the colloidal assembly to a sensing device.

Whatever the system of interest, those works illustrate that a delicate balance has to be found between the induction of specific particle-biomolecule interactions to create a significant bio-mineral interface and the preservation of the structure and activity of the biological moiety, not only during the self-assembly process but also within the resulting nanocomposite. The proper integration of natural molecules within synthetic systems therefore requires a better understanding of the effect of particle size and surface chemistry on the conformation and mobility of the biomolecule. This constitutes a first step towards the identification of the optimal conditions for creating synergy between the two systems. The task is certainly challenging and requires a close collaboration between materials chemists and biophysicists/biologists. However, this approach offers a unique possibility for achieving a controlled positioning of inorganic particles within 3D constructs that also integrate biological responsiveness.

## 5. Conclusions

The biological routes by which living organisms create elaborated hierarchical silica structures are still far from being fully understood. Model *in vitro* systems have suggested that a combination of silica formation activation via attractive electrostatic interactions and biomolecular self-assembly providing multi-scale templating is involved in the biopatterning process. These guidelines have proven useful for the synthesis of a wide diversity of artificial siliceous structures with complex morphology. Recent works indicate that it is possible to go beyond the sole structural properties of self-organized biomolecules and to take advantage of their intrinsic biological activity to design biofunctional silica nanoparticle assemblies. We strongly believe that it opens the way towards highly-tunable new materials with a wide diversity of compositions, structures and properties and, ultimately, applications.
